# *Helicobacter pylori* Infection in Rome IV Functional Dyspepsia: Association with the Epigastric Pain Syndrome Phenotype and Long-Term Response to Eradication

**DOI:** 10.3390/medicina62102040

**Published:** 2026-10-21

**Authors:** Mustafa Sadeçolak, Selma Demirbaş Yüceldi, Arda Bilgili, Mukaddes Tozlu, Ahmet Tarık Eminler, Mustafa İhsan Uslan

**Affiliations:** 1 Department of Gastroenterology, Faculty of Medicine, Hitit University, Çorum 19030, Türkiye; 2 Department of Gastroenterology, Faculty of Medicine, Sakarya University, Sakarya 54290, Türkiye; selmademirbas@yahoo.com (S.D.Y.); mukaddes_tozlu@hotmail.com (M.T.); eminler77@gmail.com (A.T.E.); iuslan@sakarya.edu.tr (M.İ.U.); 3 Department of Gastroenterology, Necip Fazil City Hospital, Kahramanmaraş 46050, Türkiye; bilgiliarda@gmail.com

**Keywords:** functional dyspepsia, *Helicobacter pylori*, Rome IV, epigastric pain syndrome, postprandial distress syndrome, *H. pylori*-associated dyspepsia, eradication therapy

## Abstract

*Background and Objectives*: Functional dyspepsia (FD) is a heterogeneous disorder of gut–brain interaction with a multifactorial pathophysiology. Although *Helicobacter pylori* infection has been implicated in FD, its relationship with Rome IV FD subgroups and the extent to which successful eradication results in sustained symptomatic improvement remain unclear. This study aimed to evaluate the distribution of *H. pylori* infection across Rome IV FD subgroups and to assess long-term symptomatic response following successful eradication. *Materials and Methods*: This prospective, single-center observational cohort study included patients with dyspeptic symptoms who underwent evaluation according to the Rome IV criteria. Adult patients who completed the Rome IV gastroduodenal symptom questionnaire and underwent upper gastrointestinal endoscopy were evaluated. Patients fulfilling the Rome IV criteria for FD without an organic cause explaining their symptoms were classified as postprandial distress syndrome (PDS), epigastric pain syndrome (EPS), or overlapping PDS–EPS. *H. pylori* status was determined histopathologically using gastric antral and corpus biopsies. *H. pylori*-positive patients received 14-day bismuth-based quadruple therapy, and eradication was assessed using a stool antigen test 8–12 weeks after treatment. Symptomatic response was evaluated at month 2, 6, and 12 using Likert scale and clinical assessment. *Results*: Of 425 patients evaluated, 244 fulfilled the Rome IV criteria for FD, and 127 (52.0%) were *H. pylori* positive. The prevalence of *H. pylori* infection differed significantly among the Rome IV FD subgroups (*p* < 0.001) and was highest in the EPS subgroup (73.4%, 58/79), compared with the PDS (37.7%, 40/106) and overlapping (49.2%, 29/59) subgroups. *H. pylori* positivity was significantly higher in EPS than in both PDS and overlapping subgroups (*p* < 0.001 and *p* = 0.003, respectively). First-line eradication rates were 96.4% (106/110) per protocol and 83.5% (106/127) by intention to treat. Symptomatic response rates following successful eradication were 43.4% (46/106), 60.4% (64/106), and 70.2% (66/94) at month 2, 6, and 12, respectively, compared with 47.0% (55/117), 63.4% (71/112), and 78.4% (80/102) in *H. pylori*-negative patients. Between-group differences in symptomatic response were not statistically significant (*p* = 0.58, 0.64, and 0.18, respectively). In the conservative sensitivity analysis, month 12 response was lower in the *H. pylori*-positive cohort (55.1% vs. 68.4%, *p* = 0.03). Sustained complete symptom resolution occurred in 25.5% (24/94) of patients after successful eradication and 19.6% (20/102) of *H. pylori*-negative patients (*p* = 0.32). *Conclusions*: *H. pylori* infection was significantly more prevalent in the EPS phenotype, supporting the biological heterogeneity of Rome IV functional dyspepsia. Sustained complete symptom resolution occurred in 25.5% after successful eradication and 19.6% in the *H. pylori*-negative cohort. Despite successful eradication in 96.4% per protocol and 83.5% by intention to treat, no clear symptomatic advantage over the *H. pylori*-negative cohort was demonstrated. Differences in phenotype distribution, treatment exposure, and follow-up completeness limit direct comparisons and preclude conclusions of equivalence or attribution of symptom resolution to eradication alone.

## 1. Introduction

Functional dyspepsia (FD) is one of the most common functional gastrointestinal disorders, with a recent systematic review and meta-analysis reporting a global pooled prevalence of 8.4%, which decreased to 6.8% when the Rome IV criteria were applied. It negatively affects quality of life and daily functioning, leading to increased healthcare utilization, reduced work productivity, and a substantial economic burden [1,2]. However, the pathogenesis of FD remains incompletely understood, and multiple mechanisms, including visceral hypersensitivity, gastroduodenal motility disturbances, low-grade inflammation, alterations in the gut–brain axis, and *Helicobacter pylori* (*H. pylori*) infection, are thought to contribute to its pathogenesis [3].

According to the Rome IV criteria, functional dyspepsia is classified into two main subgroups: postprandial distress syndrome (PDS) and epigastric pain syndrome (EPS). The substantial overlap between PDS and EPS observed with the Rome III criteria has been markedly reduced with the Rome IV criteria, allowing for the identification of more homogeneous patient groups [2]. This distinction has also raised the possibility that different FD subgroups may involve distinct pathophysiological mechanisms. In particular, the weaker association of symptoms with meals in the EPS subgroup suggests that different mechanisms may underlie the clinical manifestations of these subgroups [2].

In recent years, functional gastrointestinal disorders have increasingly been reconsidered within the framework of disorders of gut–brain interaction, with growing emphasis on the need to complement symptom-based classifications with underlying pathophysiological mechanisms. Therefore, studies investigating the distribution of *H. pylori* infection across Rome IV subgroups may contribute to a better characterization of the biological subtypes of functional dyspepsia and may help inform future classification systems [1,2,3,4].

*Helicobacter pylori* is a common infectious agent affecting approximately half of the global population and is considered one of the factors potentially involved in the pathogenesis of functional dyspepsia [5,6]. Current evidence indicates that eradication therapy in *H. pylori*-positive patients with functional dyspepsia provides a significant but modest improvement in dyspeptic symptoms. The concept of “*H. pylori*-associated dyspepsia” was introduced by the Kyoto global consensus and subsequently adopted by Rome IV, referring to sustained symptom resolution following successful *H. pylori* eradication [7].

This distinction makes it challenging to differentiate “*H. pylori*-associated dyspepsia” from “functional dyspepsia,” particularly in regions where *H. pylori* infection is endemic, and complicates terminological classification. Furthermore, establishing this distinction and confirming a diagnosis of functional dyspepsia requires a prolonged follow-up period.

However, the relationship between *H. pylori* infection and the functional dyspepsia subgroups defined by the Rome IV criteria has not been sufficiently investigated. Particularly in populations with a high prevalence of *H. pylori*, it remains unclear whether the distribution of infection differs between the EPS and PDS subgroups and whether these subgroups differ in their response to eradication therapy. Türkiye, where the prevalence of *H. pylori* has been reported to range from 65% to 82.5%, represents a suitable population in which to investigate these associations [8,9].

Therefore, characterizing the distribution of *H. pylori* infection across subgroups of patients with functional dyspepsia defined according to the Rome IV criteria may contribute to a better understanding of the biological heterogeneity of functional dyspepsia, facilitate the refinement of current terminology, and support the development of more targeted therapeutic strategies. These findings may also provide insights that could inform future revisions of the Rome classification system. The aim of this study was to evaluate the prevalence of *H. pylori* infection among functional dyspepsia subgroups classified according to the Rome IV criteria in a population with a high prevalence of *H. pylori* and to assess long-term symptomatic response following successful eradication in comparison with *H. pylori*-negative patients. In addition, we aimed to determine the association of *H. pylori* infection with the EPS and PDS subtypes and to assess the frequency of sustained complete symptom resolution after successful eradication.

## 2. Material and Methods

### 2.1. Study Population

Our study was a single-center, prospective observational cohort study that included patients diagnosed with functional dyspepsia according to the Rome IV criteria among those who underwent upper gastrointestinal endoscopy at the Gastroenterology Department of Sakarya University Faculty of Medicine, Sakarya Training and Research Hospital, between January 2023 and January 2025.

Patients aged 18 years or older with a preliminary diagnosis of functional dyspepsia who presented to our clinic with dyspeptic symptoms and were scheduled for upper gastrointestinal endoscopy completed the Rome IV gastroduodenal symptom questionnaire before the procedure, and a detailed medical history was obtained. Subsequently, upper gastrointestinal endoscopy was performed, and biopsy specimens were obtained for histopathological assessment of *H. pylori*. All endoscopic procedures were performed by gastroenterologists experienced in endoscopy. A standardized gastric biopsy protocol was used in accordance with ESGE recommendations, with two biopsies obtained from the antrum and two from the corpus, preferentially sampling areas with visible mucosal abnormalities. Biopsy specimens were evaluated using hematoxylin–eosin and Giemsa staining, with immunohistochemistry used in equivocal cases. *H. pylori* positivity was defined by the histological identification of organisms consistent with *H. pylori*. As all patients underwent endoscopy to exclude organic causes of dyspepsia, baseline *H. pylori* status was determined histologically using gastric biopsies obtained during the same procedure; a second diagnostic test was not routinely performed.

Functional dyspepsia was diagnosed according to the Rome IV criteria, and patients were classified as having postprandial distress syndrome (PDS), epigastric pain syndrome (EPS), or PDS–EPS overlap according to the Rome IV subgroup criteria.

Patients who incompletely or inadequately completed the Rome IV questionnaire or had incomplete clinical assessments; individuals younger than 18 years; pregnant or breastfeeding women; patients with organic gastrointestinal pathology on upper gastrointestinal endoscopy, including peptic ulcer disease, malignancy, or Los Angeles grade B or higher esophagitis; those with a history of diabetes mellitus, depression or anxiety, celiac disease, inflammatory bowel disease, or upper gastrointestinal surgery; regular users of nonsteroidal anti-inflammatory drugs or acetylsalicylic acid; patients with predominant symptoms suggestive of other gastrointestinal disorders, including gastroesophageal reflux disease, dysphagia, globus sensation, or irritable bowel syndrome; those who had received *H. pylori* eradication therapy within the preceding three months; and patients without informed consent were excluded from the study. In addition, to minimize the risk of false-negative results related to medications that could reduce the sensitivity of histopathological assessment, patients who had used proton pump inhibitors within two weeks or antibiotics or bismuth preparations within four weeks before endoscopy were excluded.

Before treatment, all patients received counseling on lifestyle modifications and dietary recommendations. *H. pylori*-positive patients received a 14-day bismuth-containing quadruple eradication regimen consisting of esomeprazole 40 mg twice daily, bismuth 500 mg four times daily, metronidazole 500 mg three times daily, and tetracycline 500 mg four times daily. Patients in whom eradication was unsuccessful after first-line therapy received a second-line eradication regimen consisting of esomeprazole 40 mg twice daily, bismuth 500 mg four times daily, levofloxacin 500 mg once daily, and tetracycline 500 mg four times daily. In patients who completed the treatment regimen as prescribed, eradication success was assessed 8–12 weeks after treatment using a stool *H. pylori* antigen test. Before testing, patients were required to have discontinued antibiotics, bismuth preparations, proton pump inhibitors, antacids, and histamine receptor antagonists for at least two weeks. A second diagnostic method, such as a rapid urease test, serology, or urea breath test, was not routinely used to determine *H. pylori* status. Patients who were unable to complete the 14-day eradication regimen or in whom successful eradication could not be confirmed by the stool *H. pylori* antigen test were not included in the symptomatic response analysis of patients who achieved successful eradication.

Gastroesophageal reflux symptoms were assessed separately during the clinical evaluation and symptom assessment. Heartburn was distinguished from epigastric burning based on the anatomical location of the symptom. A burning sensation perceived in the retrosternal region or extending from the epigastric to the retrosternal region was considered heartburn, whereas burning localized to the epigastric region without retrosternal extension was considered epigastric burning within the Rome IV framework. The presence of regurgitation was assessed separately and was considered a symptom supportive of gastroesophageal reflux. Patients with isolated heartburn and/or regurgitation were not included in the functional dyspepsia cohort. At this stage, symptom-based diagnostic assessment and assignment to the appropriate functional dyspepsia subgroup were determined by experienced gastroenterologists. Patients with Los Angeles grade B or higher esophagitis on endoscopic evaluation were excluded from the study, whereas grade A esophagitis alone was not considered an exclusion criterion.

In the *H. pylori*-negative group, patients with EPS-predominant symptoms received a proton pump inhibitor once daily for 4–8 weeks, whereas those with PDS-predominant symptoms received a prokinetic agent for 4–8 weeks. Patients meeting both PDS and EPS criteria remained classified as overlap; treatment was selected according to the predominant symptom and continued for 4–8 weeks.

Follow-up visits were scheduled at months 2, 6, and 12 from treatment initiation (month 0), defined as the date eradication therapy or *H. pylori*-negative medical treatment was initiated after review of the histopathology report. At each follow-up visit, symptomatic response was assessed based on the symptoms defining the functional dyspepsia subgroup to which each patient had been assigned at baseline. Patients were asked whether the symptoms corresponding to their respective subgroup remained bothersome to an extent that interfered with their daily activities, and responses were recorded using a four-point Likert-type scale. In patients with PDS or EPS, the symptoms defining the respective subgroup were evaluated, whereas in patients with the overlap phenotype, all symptoms defining both PDS and EPS were assessed. Symptomatic response was defined as a response of “strongly disagree” (Likert 1) or “disagree” (Likert 2) to the statement “My bothersome symptoms persist” for all symptoms evaluated. If a response of Likert 3 or 4 was recorded for any symptom, the patient was classified as a symptomatic non-responder. This approach was used as the operational definition of symptomatic response in our study to determine whether the symptoms defining the Rome IV phenotype remained bothersome enough to interfere with daily activities during follow-up. Thus, symptomatic response was defined as a binary endpoint based on the combined assessment of all symptoms constituting the patient’s functional dyspepsia phenotype.

Complete symptom resolution was defined as a Likert score of 1 for all relevant subgroup-defining symptoms. Among patients with successful *H. pylori* eradication who completed both visits, meeting this criterion at months 6 and 12 constituted the study’s operational definition of sustained complete symptom resolution after eradication. The same criterion of sustained complete symptom resolution (Likert 1 for all relevant symptoms at both months 6 and 12) was applied to *H. pylori*-negative patients who completed both visits, and the proportions were compared between cohorts.

The primary endpoint of the study was to compare the prevalence of *H. pylori* infection across functional dyspepsia subgroups classified according to the Rome IV criteria. The secondary endpoint was to compare symptomatic response rates at month 2, month 6, and month 12 between two patient groups: those who achieved successful *H. pylori* eradication and those who were *H. pylori*-negative at baseline. In the primary analysis, symptomatic response was evaluated among patients who completed consecutive follow-up visits; patients who missed a follow-up visit were not re-included in the primary analysis at subsequent time points.

Cochran’s Q test was used to evaluate changes in symptomatic response over time within each group. To ensure that paired analyses were performed on the same individuals, this analysis was restricted to patients with available symptomatic response data at all three follow-up time points (month 2, month 6, and month 12). Following a significant Cochran’s Q test, pairwise comparisons between time points were performed using the McNemar test.

To assess potential bias related to loss to follow-up and the evaluation of patients who achieved successful eradication, a conservative sensitivity analysis was performed including the entire baseline *H. pylori*-positive (*n* = 127) and *H. pylori*-negative (*n* = 117) cohorts. All patients were retained in their respective baseline cohorts, and available symptomatic response data at month 2, month 6, and month 12 were used. Patients without symptomatic response data at a given follow-up time point were conservatively classified as non-responders. Thus, the same approach to missing follow-up data was applied to both cohorts. In addition, to assess whether loss to follow-up was selective, patients who completed follow-up and those who did not were compared with respect to baseline age, sex, and functional dyspepsia phenotype.

### 2.2. Definitions

Functional dyspepsia was defined according to the Rome IV diagnostic criteria as the presence of at least one dyspeptic symptom during the preceding three months, with symptom onset at least six months before diagnosis, in the absence of structural disease that could explain the symptoms, including findings on upper gastrointestinal endoscopy. Dyspeptic symptoms included at least one of the following bothersome symptoms: postprandial fullness, early satiation, epigastric pain, and/or epigastric burning. The diagnosis of functional dyspepsia was established when the Rome IV diagnostic criteria were fully met and upper gastrointestinal endoscopy revealed no organic pathology that could explain the symptoms.

Patients diagnosed with functional dyspepsia were classified into three subgroups according to the Rome IV criteria: postprandial distress syndrome (PDS), epigastric pain syndrome (EPS), and PDS–EPS overlap. Among patients fulfilling the relevant criteria, those with bothersome postprandial fullness and/or early satiation at least three days per week were classified as PDS, whereas those with epigastric pain and/or epigastric burning at least one day per week were classified as EPS. Patients who fulfilled the symptom criteria for both PDS and EPS were classified as having PDS–EPS overlap.

### 2.3. Statistical Analysis

Statistical analyses were performed using SPSS version 23.0 (SPSS Inc., Chicago, IL, USA). Categorical variables were expressed as numbers and percentages, whereas continuous variables were presented as mean ± standard deviation. The independent-samples t-test was used to compare continuous variables between two groups, and Pearson’s chi-square test was used for comparisons of categorical variables. Bonferroni correction was applied for pairwise comparisons among functional dyspepsia subgroups. Cochran’s Q test was used to compare symptomatic response rates at month 2, month 6, and month 12 within the same patient group, and when a statistically significant difference was detected, pairwise comparisons were performed using the McNemar test. Effect sizes for between-group comparisons of categorical outcomes were reported as absolute differences in prevalence or symptomatic response rates, together with their 95% confidence intervals. For the three post hoc pairwise comparisons, Bonferroni correction was applied, with the threshold for statistical significance set at *p* < 0.0167. A *p*-value < 0.05 was considered statistically significant for all other analyses. Longitudinal symptomatic response was evaluated using logistic generalized estimating equations (GEE) with an exchangeable correlation structure and robust standard errors. The model included group, categorical time, a group × time interaction, and baseline FD phenotype (PDS, EPS, or overlap) as a categorical covariate to assess whether response trajectories differed between cohorts after adjustment for phenotype. A power assessment was also performed for the secondary endpoint. Based on the available sample sizes at each follow-up time point, the minimum detectable absolute between-group differences were calculated assuming a two-sided α of 0.05 and 80% power. The minimum detectable differences were approximately 18.6%, 18.3%, and 17.3% at month 2, month 6, and month 12, respectively.

## 3. Results

**Figure 1:**
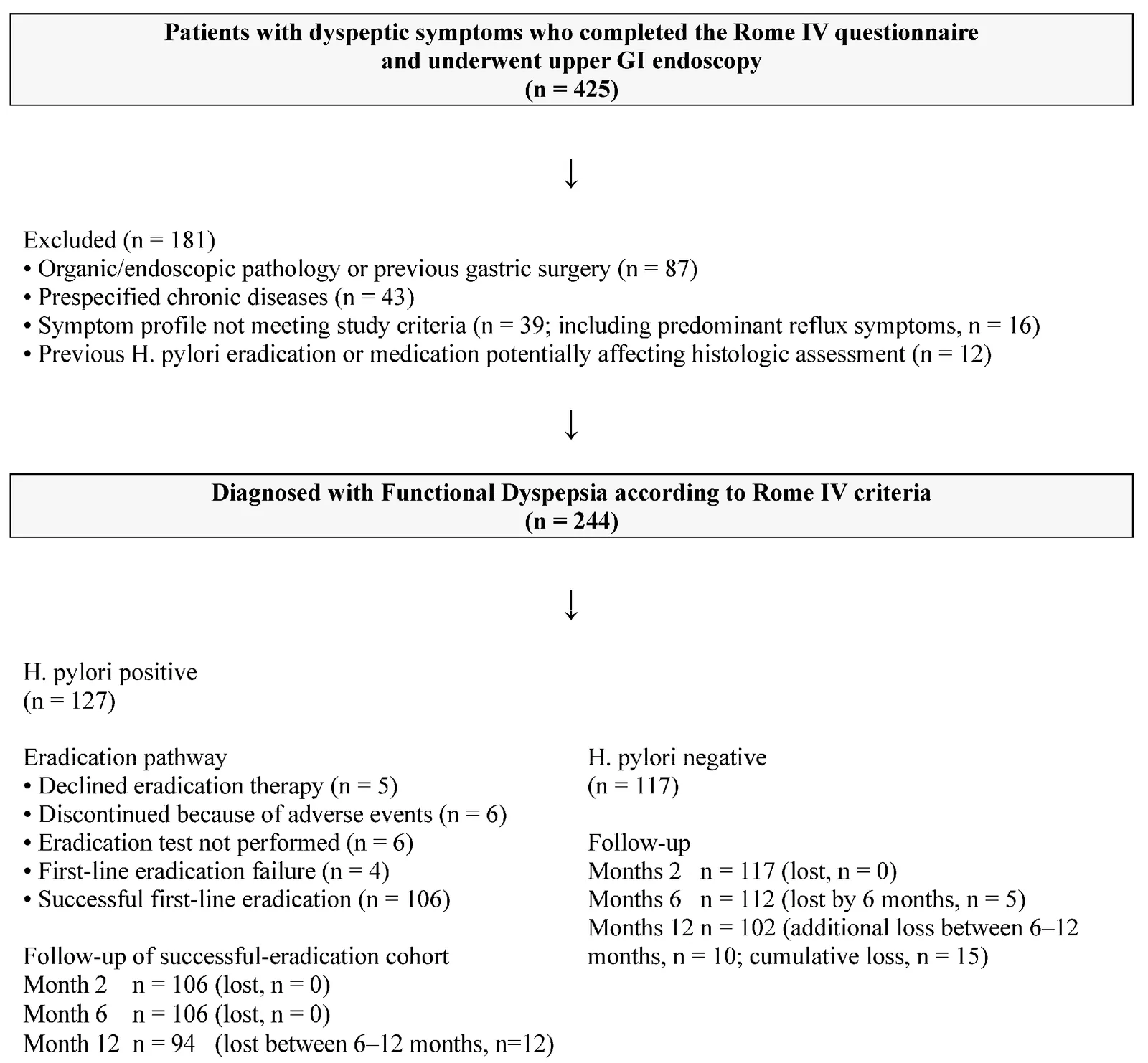
Flow diagram of the study population, exclusions, eradication pathway, and follow-up.

A total of 425 patients who completed the Rome IV gastroduodenal symptom questionnaire and underwent upper gastrointestinal endoscopy for dyspeptic symptoms were assessed for eligibility. Of these, 181 patients were excluded because they did not meet the study criteria. Eighty-seven patients were excluded because upper gastrointestinal endoscopy revealed peptic ulcer disease, malignancy, Los Angeles (LA) grade B or higher esophagitis, or anatomical changes related to previous gastric surgery. An additional 43 patients were excluded because of the previously specified chronic diseases. Thirty-nine patients were excluded because their symptom profiles were not compatible with the study criteria; among these, 16 had predominant gastroesophageal reflux symptoms, including regurgitation and/or retrosternal burning (heartburn). In addition, 12 patients were excluded because of previous *H. pylori* eradication therapy or the use of medications that could affect the histopathological assessment of *H. pylori*. After all exclusions, 244 patients were included in the final study cohort. Los Angeles grade A esophagitis was identified in three patients (1.2%; 3/244) in the final cohort (The study flow diagram is presented in Figure 1).

The mean age of patients diagnosed with functional dyspepsia according to the Rome IV criteria was 39.8 ± 13.3 years; 61.1% (149/244) were female and 38.9% (95/244) were male. Regarding functional dyspepsia subgroups, 43.4% (106/244) of patients were classified as having postprandial distress syndrome (PDS), 32.4% (79/244) as having epigastric pain syndrome (EPS), and 24.2% (59/244) as having PDS–EPS overlap. Histopathological examination revealed *H. pylori* positivity in 52.0% (127/244) of the 244 patients diagnosed with functional dyspepsia. Among *H. pylori*-positive patients, 59.1% (75/127) were female, and the mean age was 39.8 ± 12.9 years. Among the 117 *H. pylori*-negative patients, 63.2% (74/117) were female, and the mean age was 39.9 ± 13.7 years. There were no statistically significant differences between the *H. pylori*-positive and *H. pylori*-negative groups in terms of age (*p* = 0.95) or sex distribution (*p* = 0.51). Smoking was reported in 26.8% (*n* = 34) of *H. pylori*-positive patients and 24.8% (*n* = 29) of *H. pylori*-negative patients. Social alcohol consumption was reported in 16.5% (*n* = 21) and 12.8% (*n* = 15) of patients, respectively.

Among the 127 *H. pylori*-positive patients, 45.7% (*n* = 58) were classified as EPS, 31.5% (*n* = 40) as PDS, and 22.8% (*n* = 29) as PDS–EPS overlap. In contrast, among the 117 *H. pylori*-negative patients, 56.4% (*n* = 66) were classified as PDS, 17.9% (*n* = 21) as EPS, and 25.6% (*n* = 30) as PDS–EPS overlap. The prevalence of *H. pylori* infection differed significantly across the functional dyspepsia subgroups (*p* < 0.001). The prevalence of *H. pylori* infection was 73.4% (58/79) in the EPS subgroup, 37.7% (40/106) in the PDS subgroup, and 49.2% (29/59) in the PDS–EPS overlap subgroup. *H. pylori* prevalence was significantly higher in the EPS subgroup than in the PDS subgroup (95% CI for the absolute difference, 22.3–49.1%; *p* < 0.001) and the PDS–EPS overlap subgroup (95% CI for the absolute difference, 8.2–40.3%; *p* = 0.003). The difference between the PDS and PDS–EPS overlap subgroups was not statistically significant (absolute difference, −11.4%; 95% CI, −27.2% to 4.3%; *p* = 0.15) (the demographic and clinical characteristics of the study population are presented in Table 1).

Multivariable logistic regression was performed with baseline *H. pylori* positivity as the dependent variable and FD phenotype, age, sex, smoking, and alcohol consumption as explanatory variables. After adjustment for age, sex, smoking, and alcohol consumption, EPS remained associated with *H. pylori* positivity compared with PDS (aOR, 4.83; 95% CI, 2.53–9.21; *p* < 0.001) and overlap (aOR, 3.03; 95% CI, 1.47–6.24; *p* = 0.003).

Of the 127 *H. pylori*-positive patients, five declined eradication therapy, while six discontinued treatment because of adverse effects. The remaining 116 patients completed the eradication regimen. During follow-up, eradication success was assessed in 110 of these 116 patients using the stool *H. pylori* antigen test. First-line eradication rates were 96.4% (106/110) per protocol and 83.5% (106/127) by intention to treat. The five patients who declined eradication therapy and the six patients who were unable to complete treatment because of adverse effects received a standard-dose proton pump inhibitor for four weeks, whereas the six patients in whom eradication status could not be assessed received no additional treatment. The four patients in whom first-line eradication therapy failed received second-line eradication therapy, with successful eradication achieved in all four patients.

During follow-up, additional rescue treatments were administered for persistent or recurrent dyspeptic symptoms. Among patients who achieved successful *H. pylori* eradication, 26 (24.5%; 26/106) received a proton pump inhibitor once daily for four weeks at month 2, and 12 (11.3%; 12/106) received the same treatment at month 6. In the *H. pylori*-negative cohort, 32 patients (27.4%; 32/117) at month 2 and 8 patients (7.1%; 8/112) at month 6 received an additional four-week course of rescue therapy. Treatment was selected according to the functional dyspepsia phenotype, with a proton pump inhibitor once daily for patients with EPS and a prokinetic agent for those with PDS. No additional rescue treatment was administered in either cohort at month 12. Initial and rescue treatments in the *H. pylori*-negative cohort are summarized in Supplementary Table S1.

In the analysis group comprising patients who achieved successful *H. pylori* eradication and *H. pylori*-negative patients, symptomatic response rates at month 2, month 6, and month 12 were 45.3% (101/223), 61.9% (135/218), and 74.5% (146/196), respectively. Among patients who achieved successful *H. pylori* eradication, symptomatic response rates at month 2, month 6, and month 12 were 43.4% (46/106), 60.4% (64/106), and 70.2% (66/94), respectively. Among *H. pylori*-negative patients, symptomatic response rates were 47.0% (55/117), 63.4% (71/112), and 78.4% (80/102) at month 2, month 6, and month 12, respectively. Of the 21 *H. pylori*-positive patients who were not included in the successful-eradication cohort after first-line treatment, 15 attended the month 2 follow-up visit, 9 attended the month 6 visit, and 4 attended the month 12 visit. Symptomatic response rates among these patients were 33.3% (5/15), 55.6% (5/9), and 100% (4/4), respectively.

Symptomatic response rates among *H. pylori*-positive patients who achieved successful eradication and *H. pylori*-negative patients were 43.4% (46/106) and 47.0% (55/117), respectively, at month 2 (absolute difference, −3.6%; 95% CI, −16.7% to 9.5%; *p* = 0.58); 60.4% (64/106) and 63.4% (71/112), respectively, at month 6 (absolute difference, −3.0%; 95% CI, −15.9% to 9.9%; *p* = 0.64); and 70.2% (66/94) and 78.4% (80/102), respectively, at month 12 (absolute difference, −8.2%; 95% CI, −20.4% to 4.0%; *p* = 0.18). No statistically significant differences between the groups were observed at any follow-up time point (Symptomatic response rates during follow-up are presented in Table 2).

All 106 patients who achieved successful eradication completed the month 2 and month 6 follow-up visits, whereas 11.3% (12/106) did not attend the month 12 follow-up visit. In the *H. pylori*-negative group, 4.3% (5/117) of patients did not attend the month 6 follow-up visit, and an additional 8.9% (10/112) did not attend the month 12 follow-up visit. No statistically significant differences in baseline age or sex were observed between patients who completed follow-up and those who did not (age, *p* = 0.65; sex, *p* = 0.52). Similarly, the baseline distribution of functional dyspepsia phenotypes was comparable between patients who completed and those who did not complete the 12-month follow-up in both the successful-eradication cohort and the *H. pylori*-negative cohort (*p* = 0.86).

The 94 patients in the successful-eradication cohort and 102 *H. pylori*-negative patients with available symptomatic response data at all three follow-up visits were included in the within-group longitudinal analysis. In the successful-eradication cohort, symptomatic response rates at month 2, month 6, and month 12 were 40.4% (38/94), 57.4% (54/94), and 70.2% (66/94), respectively, showing a significant increase over time (Cochran’s Q = 38.19; *p* < 0.001). Similarly, in the *H. pylori*-negative cohort, symptomatic response rates were 42.2% (43/102), 63.7% (65/102), and 78.4% (80/102), respectively, with a significant increase over time (Cochran’s Q = 49.48; *p* < 0.001). In Bonferroni-adjusted post hoc McNemar analyses, significant differences were observed for all comparisons between month 2 and month 6, month 2 and month 12, and month 6 and month 12 in both cohorts (successful-eradication cohort: *p* < 0.001, *p* < 0.001, and *p* = 0.006, respectively; *H. pylori*-negative cohort: *p* < 0.001, *p* < 0.001, and *p* = 0.001, respectively).

In the conservative sensitivity analysis, in which the entire baseline cohorts were retained and patients without follow-up data were classified as non-responders, symptomatic response rates in the *H. pylori*-positive and *H. pylori*-negative cohorts were 40.2% (51/127) and 47.0% (55/117), respectively, at month 2 (absolute difference, −6.9%; 95% CI, −19.3% to 5.6%; *p* = 0.28); 54.3% (69/127) and 60.7% (71/117), respectively, at month 6 (absolute difference, −6.4%; 95% CI, −18.7% to 6.0%; *p* = 0.31); and 55.1% (70/127) and 68.4% (80/117), respectively, at month 12 (absolute difference, −13.3%; 95% CI, −25.3% to −1.2%; *p* = 0.03). No statistically significant difference was observed at the first two time points, whereas symptomatic response at month 12 was significantly higher in the *H. pylori*-negative cohort. The GEE analysis included 223 patients and 637 observations. Symptomatic response rates by baseline FD phenotype within each cohort are presented in Table 3. After adjustment for phenotype, the group × time interaction remained non-significant (Wald χ^2^ = 1.07; df = 2; *p* = 0.59). In the LOCF sensitivity analysis, which carried the last available response forward and retained patients without any follow-up data as non-responders, month 12 response rates were 66.9% (85/127) and 76.1% (89/117), respectively (*p* = 0.115).

Among the 94 patients who achieved successful *H. pylori* eradication and completed both the month 6 and month 12 follow-up visits, 24 (25.5%) had complete symptom resolution (Likert = 1) at both time points. These 24 patients represented 22.6% of the primary successful-eradication cohort (24/106) and 18.9% of the baseline *H. pylori*-positive cohort (24/127). Thirty-three patients were not included in this classification: 21 were outside the primary successful-eradication cohort, and 12 lacked month 12 follow-up data. In the *H. pylori*-negative cohort, 20 of 102 patients (19.6%) who completed both visits met the same criterion for sustained complete symptom resolution. One of these 24 patients (4.2%) received rescue PPI therapy at month 2 but still met the operational definition, with complete symptom resolution (Likert 1) at both months 6 and 12. The difference between cohorts was not statistically significant (25.5% vs. 19.6%; *p* = 0.32). After excluding the 24 patients who met the study’s operational definition of sustained complete symptom resolution after successful eradication (nine EPS, eight PDS, and seven overlap), 220 patients remained. Baseline *H. pylori* positivity was 70.0% in EPS, 32.7% in PDS, and 42.3% in overlap (overall *p* < 0.001). The adjusted association persisted for EPS versus PDS (aOR, 5.00; 95% CI, 2.56–9.79; *p* < 0.001) and overlap (aOR, 3.31; 95% CI, 1.55–7.07; *p* = 0.002).

## 4. Discussion

In our study, we evaluated the distribution of *H. pylori* infection across functional dyspepsia subgroups in patients diagnosed according to the Rome IV criteria and symptomatic response rates following eradication therapy. The main finding of our study was that *H. pylori* infection was unevenly distributed across the Rome IV functional dyspepsia subgroups, with the prevalence of infection being significantly higher in the EPS group than in the PDS and PDS–EPS overlap groups. In contrast, PDS was the most common functional dyspepsia subgroup in the overall patient population and among *H. pylori*-negative patients. Symptomatic response increased over time in both cohorts, and neither the primary nor the LOCF analysis demonstrated a statistically significant between-group difference. Although the conservative sensitivity analysis showed a lower month 12 response in the *H. pylori*-positive cohort, this difference was not maintained under LOCF, highlighting the influence of missing-data assumptions. Together, these findings support an association between *H. pylori* infection and the EPS phenotype but do not demonstrate a clear long-term symptomatic advantage following eradication over the *H. pylori*-negative cohort; differences between cohorts limit causal interpretation.

Functional dyspepsia is one of the common gastrointestinal disorders and has a substantial impact on quality of life and healthcare utilization. A recent comprehensive systematic review and meta-analysis reported a global prevalence of functional dyspepsia of 8.4%, which decreased to 6.8% when the more stringent Rome IV diagnostic criteria were applied [10]. The application of the Rome IV criteria aims to define functional dyspepsia subgroups based on more specific symptom characteristics and thereby establish more homogeneous patient groups. In our study, of the 425 patients evaluated for dyspeptic symptoms and who underwent upper gastrointestinal endoscopy, 244 fulfilled the Rome IV criteria and were classified as having functional dyspepsia. Among patients diagnosed with functional dyspepsia, 43.4% were classified as PDS, 32.4% as EPS, and 24.2% as PDS–EPS overlap. Population-based studies using the Rome IV criteria have reported PDS as the most common functional dyspepsia subgroup, and the predominance of PDS in our study is generally consistent with these findings [11]. Similarly, the relatively young mean age and the predominance of female patients in our functional dyspepsia cohort are consistent with the literature. However, because our study was not a population-based prevalence study and included symptomatic patients undergoing upper gastrointestinal endoscopy, direct comparison of the distribution of functional dyspepsia and its subgroups in our patient population with population-based prevalence data may not be appropriate [12].

One of the notable findings of our study was that *H. pylori* infection was unevenly distributed across the Rome IV functional dyspepsia subgroups, with the prevalence of infection being significantly higher in the EPS group (73.4%) than in the PDS (37.7%) and PDS–EPS overlap (49.2%) groups. The lower overall *H. pylori* prevalence in our cohort (52.0%) compared with previous reports from Türkiye (65–82.5%) may reflect the exclusion of peptic ulcer disease and recent eradication therapy, together with regional and temporal differences between study populations [8,9]. However, the available evidence regarding the association between *H. pylori* infection and functional dyspepsia subgroups is limited and not entirely consistent. A study using the Rome III criteria reported that *H. pylori* infection may influence the pathophysiological characteristics of functional dyspepsia [13], whereas another study showed that symptomatic benefit following successful eradication was more pronounced particularly in patients with EPS [14]. These findings support a potential pathophysiological association between *H. pylori* infection and the EPS subgroup in patients with functional dyspepsia. In contrast, some studies have suggested that the presence of *H. pylori* does not reliably distinguish between the EPS and PDS subgroups and that symptom-based subgroup classification may be insufficient to predict the pathogenetic role of the infection [15].

The significant association between EPS and *H. pylori* observed in our study suggests that chronic gastric inflammation related to the infection may contribute to the development of a pain- and burning-predominant phenotype. The finding that *H. pylori*-positive patients with functional dyspepsia have a lower gastric sensory threshold and higher levels of sensory neuropeptides suggests a potential link between the infection and visceral hypersensitivity [16]. However, the high prevalence of *H. pylori* infection among asymptomatic individuals and the potentially multifactorial pathophysiology of functional dyspepsia preclude interpreting the association observed in our study as evidence of causality. Our findings indicate that, particularly in populations with a high prevalence of *H. pylori*, the association between the EPS phenotype and infection should be further investigated in larger, multicenter studies using the Rome IV criteria.

In the primary analysis based on patients who achieved successful eradication, no statistically significant differences in symptomatic response rates were observed between the *H. pylori*-positive and *H. pylori*-negative cohorts at month 2, month 6, or month 12. However, in the sensitivity analysis that included all patients in the baseline *H. pylori*-positive and *H. pylori*-negative cohorts and conservatively classified patients without follow-up data as non-responders, the findings remained unchanged at month 2 and month 6, whereas at month 12, the symptomatic response rate was significantly higher in the *H. pylori*-negative cohort (68.4% vs. 55.1%; *p* = 0.034). Most patients lost after month 6 had previously responded (10/12 in the successful-eradication cohort and 6/10 in the negative cohort), and missing responses were more frequent in the baseline positive cohort (29/127 vs. 15/117). The month 12 difference was no longer statistically significant under LOCF, suggesting that the conservative result may partly reflect unequal follow-up loss rather than a difference in symptomatic outcomes.

Nevertheless, our results showed that symptomatic response rates increased over time among *H. pylori*-positive patients who achieved successful eradication. A similar increase in symptomatic improvement over time was observed in the *H. pylori*-negative group, and no statistically significant differences in symptomatic response rates were found between the two groups at month 2, month 6, or month 12. This finding suggests that symptomatic improvement in functional dyspepsia is not attributable solely to *H. pylori* eradication and supports the multifactorial nature of the disorder. Current guidelines and meta-analyses indicate that eradication therapy provides symptomatic benefit in *H. pylori*-positive patients with functional dyspepsia, although the absolute magnitude of this benefit is limited. In the meta-analysis by Ford et al., eradication therapy significantly improved long-term symptoms compared with placebo, although the magnitude of the symptomatic benefit was reported to be modest [5]. Similarly, Kang et al. reported symptomatic benefit from eradication in both high (≥50%; RR = 1.17, 95% CI: 1.06–1.29) and low (<50%; RR = 1.21, 95% CI: 1.02–1.44) *H. pylori* prevalence regions, whereas the Asian subgroup did not reach statistical significance (RR = 1.14, 95% CI: 0.99–1.33) [6]. These findings suggest that functional dyspepsia cannot be explained by a single pathophysiological mechanism and that multiple factors may contribute to changes in symptoms over time. Functional dyspepsia is currently regarded as a heterogeneous disorder involving the combined effects of impaired gastric motility, visceral hypersensitivity, low-grade duodenal inflammation, impaired mucosal barrier integrity, alterations in the gastrointestinal microbiota, and dysregulation of gut–brain interactions. Therefore, *H. pylori* infection may represent only one of several factors contributing to symptom development in a specific subgroup of patients [1]. The similar improvement rates observed among *H. pylori*-negative patients in our study may likewise reflect the combined effects of these different mechanisms together with the natural course of functional dyspepsia.

Another important aspect of our study is the evaluation of the association between *H. pylori* and Rome IV functional dyspepsia subgroups and the potential contribution of our findings to the ongoing terminological discussion. According to the Rome IV criteria, *H. pylori*-positive patients whose symptoms completely resolve for at least six months following successful eradication are considered to have “*H. pylori*-associated dyspepsia,” whereas those with persistent symptoms are considered to have functional dyspepsia [17,18]. However, distinguishing between these two clinical entities may not always be straightforward in populations with a high prevalence of *H. pylori*. In our study, although *H. pylori* infection was markedly more frequent in the EPS subgroup, symptomatic response following eradication did not differ significantly from that observed in *H. pylori*-negative patients, suggesting that the presence of *H. pylori* does not represent a common pathogenetic mechanism in all cases of functional dyspepsia. Nevertheless, the strong association observed with the EPS phenotype may be regarded as a hypothesis-generating finding suggesting that *H. pylori* infection may play a more prominent role in a specific biological subtype of functional dyspepsia. Sustained complete symptom resolution occurred in 25.5% of evaluable patients following successful eradication and 19.6% of *H. pylori*-negative patients, without a statistically significant difference. Its occurrence in both cohorts limits attribution of sustained resolution to eradication alone.

From the Rome V perspective, the concept of “disorders of gut–brain interaction” has increasingly been approached through a more precise and mechanism-based framework, with greater emphasis on phenotyping these disorders not only according to symptoms but also according to underlying pathophysiological mechanisms and biological markers [19]. In this context, our findings provide hypothesis-generating data that may contribute to future mechanism-based phenotyping approaches in functional dyspepsia, integrating biological markers such as *H. pylori* alongside symptom-based subgroups [20]. A study investigating a faecal microRNA marker in *H. pylori* gastritis suggests a potential avenue for further research. Whether such markers can distinguish FD phenotypes or predict symptomatic response requires further investigation [21]. Additionally, Rome V revises the subgroup definitions, including a lower symptom-frequency threshold for PDS and greater emphasis on the relationship of symptoms to meals. Our cohort was classified using Rome IV; therefore, the observed phenotype distributions should not be directly extrapolated to Rome V [22].

Our study also has several important strengths. To our knowledge, it is among the first studies conducted in a region with a high prevalence of *H. pylori* to evaluate both the distribution of *H. pylori* across functional dyspepsia subgroups classified according to the Rome IV criteria and long-term symptomatic response following eradication. The exclusion of organic pathology in all patients by upper gastrointestinal endoscopy and histopathological examination, the use of a standardized eradication regimen, and follow-up for up to 12 months strengthen the methodological robustness of our study. Furthermore, evaluating not only the presence of *H. pylori* infection but also its association with Rome IV subgroups provides original data that may contribute to a better understanding of the biological phenotypes of functional dyspepsia.

Our study has several limitations. First, as a single-center observational study, the generalizability of our findings may be limited. In addition, symptom assessment was based on a patient-reported Likert scale, which may be subject to subjective reporting bias. Nevertheless, given that the primary aim of our study was to evaluate the distribution of *H. pylori* infection across functional dyspepsia subgroups and symptomatic response following eradication, our findings should be confirmed in larger, multicenter prospective studies. Although the primary analysis based on patients who achieved successful eradication was susceptible to selection bias and bias related to loss to follow-up, a conservative sensitivity analysis including the entire baseline cohorts was performed to assess the potential impact of these sources of bias.

Another limitation of our study is that heartburn was distinguished from epigastric burning based on symptom location and the presence of regurgitation. Because these symptoms may overlap in clinical practice, excluding patients with predominant heartburn and/or regurgitation may have introduced some degree of selection or classification bias, particularly in the EPS subgroup, in which epigastric burning is a defining symptom. This should be considered when interpreting the observed association between EPS and *H. pylori* infection. In addition, three patients in the final cohort had Los Angeles grade A esophagitis, indicating that some overlap between mild reflux findings and functional dyspepsia could not be completely excluded. Finally, reflux symptoms were not assessed using a standardized symptom scale or ambulatory reflux monitoring, which may have limited the accuracy of this distinction.

Another limitation of our study is that *H. pylori* status was determined solely by histopathological examination and was not confirmed using a second diagnostic method. Although recommended washout periods for PPIs, antibiotics, and bismuth were applied before endoscopy and biopsies were obtained from both the antrum and corpus, the use of histology alone cannot completely exclude the possibility of false-negative results in patients with low bacterial density. Such misclassification could result in truly *H. pylori*-positive patients being included in the negative group, potentially reducing the observed differences between the groups. However, if the extent of misclassification differed across functional dyspepsia subgroups, the direction of the resulting bias would be difficult to predict.

Because the two cohorts had different treatment exposures at baseline and during follow-up, the comparison of symptomatic response should not be interpreted as an analysis of the isolated effect of *H. pylori* eradication. Specifically, *H. pylori*-positive patients with PDS did not receive prokinetics, whereas *H. pylori*-negative patients with PDS received prokinetic therapy, which may have influenced symptomatic outcomes. Rather, our findings should be interpreted as a comparison of the long-term symptomatic course of two prospectively followed observational cohorts managed differently according to their clinical needs. In addition, although no statistically significant between-group differences were observed in the secondary analysis, the power assessment and the width of the 95% confidence intervals indicate that smaller but clinically meaningful differences cannot be excluded. Therefore, these findings should not be interpreted as evidence of equivalence in symptomatic response between the two cohorts.

In our study, patients with a diagnosis of anxiety or depression were excluded, and smoking and alcohol use were assessed. However, psychological status was not systematically evaluated using validated scales, and other potential confounding variables, such as socioeconomic status, were not recorded. Therefore, the potential effects of unmeasured confounders, particularly subclinical anxiety and depressive symptoms, on functional dyspepsia subgroup distribution and symptomatic response cannot be completely excluded. Although the longitudinal symptomatic response model was adjusted for baseline FD phenotype, residual confounding from other measured and unmeasured factors cannot be excluded.

## 5. Conclusions

In conclusion, *H. pylori* infection is not uniformly distributed across Rome IV functional dyspepsia subgroups and is significantly more prevalent in the EPS subgroup. Despite first-line eradication rates of 96.4% per protocol and 83.5% by intention to treat, a clear symptomatic advantage over *H. pylori*-negative patients was not demonstrated. Differences between the cohorts and incomplete follow-up limit this comparison and do not establish equivalence. Sustained complete symptom resolution occurred in 25.5% of evaluable patients after successful eradication and 19.6% of *H. pylori*-negative patients, limiting attribution to eradication alone. Our findings support the possibility that *H. pylori* infection may play a more prominent role in specific biological subtypes of functional dyspepsia and provide hypothesis-generating data that may contribute to the mechanism-based phenotyping approach envisioned in the Rome V framework. Large-scale, multicenter prospective studies involving patient populations from different geographical regions are needed to confirm these findings.

## Figures and Tables

**Table 1:** Baseline characteristics of patients with functional dyspepsia according to *H. pylori* status.

Variable	*H. pylori* (+) *n* = 127	*H. pylori* (−) *n* = 117	*p* Value
Age (years)	39.8 ± 12.9	39.9 ± 13.7	0.95
Sex			0.51
Female, *n* (%)	75 (59.1)	74 (63.2)	
Male, *n* (%)	52 (40.9)	43 (36.8)	
Smoking, *n* (%)	34 (26.8)	29 (24.8)	0.72
Social Alcohol Consumption, *n* (%)	21 (16.5)	15 (12.8)	0.41
Rome IV subgroup			<0.001
PDS, *n* (%)	40 (31.5)	66 (56.4)	
EPS, *n* (%)	58 (45.7)	21 (17.9)	
Overlap, *n* (%)	29 (22.8)	30 (25.6)	

Overall *p* value was calculated using the Pearson chi-square test. Post hoc pairwise comparisons with Bonferroni correction: EPS vs. PDS, *p* < 0.001; EPS vs. overlap, *p* = 0.003; PDS vs. overlap, *p* = 0.154. *H. pylori*, *Helicobacter pylori*; PDS, Postprandial Distress Syndrome; EPS, Epigastric Pain Syndrome.

**Table 2:** Clinical outcomes after *H. pylori* eradication.

Outcome	*H. pylori* (+)	*H. pylori* (−)	*p* Value
Eradication completed *n* (%)	116/127 (91.3%)	—	—
Stool antigen test *n* (%)	110/116 (94.8%)	—	—
Successful eradication *n* (%)	106/110 (96.4%)	—	—
Primary analysis			
Symptomatic response (Month 2), *n* (%)	46/106 (43.4%) *	55/117 (47.0%)	0.58
Symptomatic response (Month 6), *n* (%)	64/106 (60.4%) *	71/112 (63.4%)	0.64
Symptomatic response (Month 12), *n* (%)	66/94 (70.2%) *	80/102 (78.4%)	0.18
Conservative sensitivity analysis **			
Symptomatic response (Month 2), *n* (%)	51/127 (40.2)	55/117 (47.0)	0.28
Symptomatic response (Month 6), *n* (%)	69/127 (54.3)	71/117 (60.7)	0.31
Symptomatic response (Month 12), *n* (%)	70/127 (55.1)	80/117 (68.4)	0.03
LOCF sensitivity analysis ***			
Symptomatic response (Month 12), *n* (%)	85/127 (66.9)	89/117 (76.1)	0.115

* Primary symptomatic response analysis included patients with successful *H. pylori* eradication (*n* = 106 at months 2 and 6; *n* = 94 at month 12). ** Includes the entire baseline cohorts; missing responses were classified as non-response. *** Last available responses were carried forward; patients without any follow-up response data were retained as non-responders.

**Table 3:** Symptomatic response by baseline functional dyspepsia phenotype within each cohort.

Cohort	Phenotype	Month 2, *n*/N (%)	Month 6, *n*/N (%)	Month 12, *n*/N (%)
*H. pylori*-positive patients excluded from the primary analysis	EPS	4/9 (44.4)	3/6 (50.0)	2/2 (100.0)
	PDS	1/3 (33.3)	1/1 (100.0)	1/1 (100.0)
	Overlap	0/3 (0.0)	1/2 (50.0)	1/1 (100.0)
Successful *H. pylori* eradication	EPS	21/46 (45.7)	28/46 (60.9)	28/41 (68.3)
	PDS	15/36 (41.7)	21/36 (58.3)	23/32 (71.9)
	Overlap	10/24 (41.7)	15/24 (62.5)	15/21 (71.4)
*H. pylori*-negative	EPS	10/21 (47.6)	12/20 (60.0)	13/17 (76.5)
	PDS	33/66 (50.0)	40/64 (62.5)	45/58 (77.6)
	Overlap	12/30 (40.0)	19/28 (67.9)	22/27 (81.5)

EPS, epigastric pain syndrome; PDS, postprandial distress syndrome; *H. pylori*, *Helicobacter pylori*; *n*/N, number of responders/number of patients assessed at each visit.

## Data Availability

The data supporting the findings of this study are available from the corresponding author upon reasonable request. The data are not publicly available due to patient privacy and ethical restrictions.

## Supplementary Materials

The following supporting information can be downloaded at https://www.mdpi.com/article/10.3390/medicina62102040/s1. Table S1: Initial and rescue treatments by phenotype in the *H. pylori*-negative cohort.
