# Supplementary material for: Helicobacter pylori Infection in Rome IV Functional Dyspepsia: Association with the Epigastric Pain Syndrome Phenotype and Long-Term Response to Eradication
Source: Medicina (Kaunas). 2026 Oct 21;62(10):2040. doi: 10.3390/medicina62102040 (PMC13706855; doi:10.3390/medicina62102040)
Supplement: Supplementary file 1 [file medicina-62-02040-s001.zip › medicina-4535214-supplementary.pdf]

Supplementary Table S1. Initial and rescue treatments by phenotype in the H. pylori-negative cohort

Phenotype	Initial treatment		Month 2 rescue		Month 6 rescue	
	PPI, n	Prokinetic, n	PPI, n	Prokinetic, n	PPI, n	Prokinetic, n
EPS	21	0	5	0	2	0
PDS	0	66	0	18	0	5
Overlap	18	12	6	3	1	0
**Total**	**39**	**78**	**11**	**21**	**3**	**5**

Values are numbers of patients receiving each drug class at the indicated time point.

Abbreviations: EPS, epigastric pain syndrome; PDS, postprandial distress syndrome; PPI, proton pump inhibitor; H. pylori, Helicobacter pylori
